# How Tumor Cells Acquire Resistance to Kinase Inhibitors

**DOI:** 10.1371/journal.pmed.0020074

**Published:** 2005-02-22

Acquired resistance to chemotherapy is a major obstacle to successful cancer treatment. Understanding the mechanisms by which tumors become resistant to a particular agent is key to identifying new drugs or combination regimens.

Kinases are signaling molecules that control many aspects of cell behavior, including cell proliferation, i.e., whether and how fast cells divide. Abnormally active kinases promoting tumor growth are found in many cancers and are a focus of rational cancer drug design. One target for kinase inhibitors is the epidermal growth factor receptor (EGFR). Two EGFR inhibitors, gefitinib and erlotinib, showed therapeutic benefits in a subset of patients with non-small cell lung cancer. Recent work has helped us understand why some patients respond and some don't: responsive tumors usually harbor activating mutations in the EGFR gene, which somehow make the tumors sensitive to treatment. Nearly all patients whose tumors initially respond to EGFR inhibitors, however, eventually become resistant to the drugs and progress despite continued therapy.

William Pao and colleagues examined tumors from six patients with non-small cell lung cancer who initially responded to gefitinib or erlotinib but subsequently relapsed. Tumors from all six patients carried activating mutations in the EGFR gene. In addition, in three out of the six cases, the resistant tumor cells carried an identical second mutation in the EGFR gene. Whereas the activating mutation was present in tumor cells before treatment with erlotinib or gefitinib, the second mutation was not found in pre-treatment biopsies from these patients, nor in over 150 lung cancer samples from patients who had not been treated with either drug. Additional cell culture studies supported the notion that the secondary mutation causes resistance to gefitinib or erlotinib. It is clear, though, that this is only one mechanism of resistance, because in the three other cases resistance occurred in the absence of the second mutation. What caused the resistance in those tumors is not known.

All kinases share some common features, and a resistance mutation very similar to the one identified here has also been found in other kinase genes from tumors with acquired resistance to imatinib, another kinase inhibitor. As Gary Gilliland and colleagues point out in an accompanying Perspective (DOI: 10.1371/journal.pmed.0020075), the initial identification three years ago of resistance mutations against imatinib led to the rapid development of alternative kinase inhibitors that work even against tumors with the resistance mutation. Similarly, the results by Pao and colleagues should help researchers develop second generation drugs for lung cancer.

**Figure:**
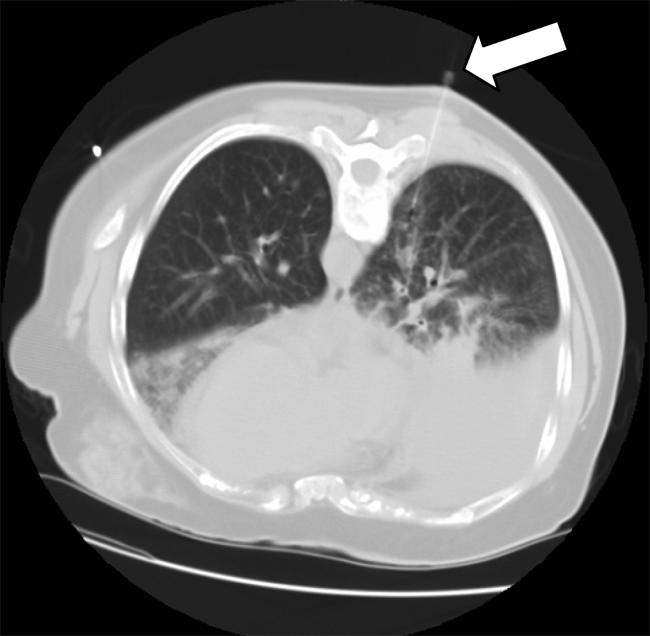
Re-biopsy of progressing lung lesion

